# New Insights on *Gordonia alkanivorans* Strain 1B Surface-Active Biomolecules: Gordofactin Properties

**DOI:** 10.3390/molecules30010001

**Published:** 2024-12-24

**Authors:** João Tavares, Susana M. Paixão, Tiago P. Silva, Luís Alves

**Affiliations:** 1 Unidade de Bioenergia e Biorrefinarias, LNEG—Laboratório Nacional de Energia e Geologia, Estrada do Paço do Lumiar 22, 1649-038 Lisboa, Portugal; 2 RCM2+–Centro de Investigação em Gestão de Ativos e Engenharia de Sistemas, Universidade Lusófona, Campo Grande 376, 1749-024 Lisboa, Portugal

**Keywords:** gordofactin, biosurfactant/bioemulsifier, *Gordonia alkanivorans* strain 1B, critical micelle concentration, emulsifying activity, antioxidant activity, antimicrobial activity

## Abstract

Biosurfactants/bioemulsifiers (BSs/BEs) can be defined as surface-active biomolecules produced by microorganisms with a broad range of applications. In recent years, due to their unique properties like biodegradability, specificity, low toxicity, and relative ease of preparation, these biomolecules have attracted wide interest as an eco-friendly alternative for several industrial sectors, escalating global microbial BS/BE market growth. Recently, *Gordonia alkanivorans* strain 1B, a bacterium with significant biotechnological potential, well known for its biodesulfurizing properties, carotenoid production, and broad catabolic range, was described as a BS/BE producer. This study focuses on the characterization of the properties of the lipoglycopeptide BSs/BEs produced by strain 1B, henceforth referred to as gordofactin, to better understand its potential and future applications. Strain 1B was cultivated in a chemostat using fructose as a carbon source to stimulate gordofactin production, and different purification methods were tested. The most purified sample, designated as extracted gordofactin, after lyophilization, presented a specific emulsifying activity of 9.5 U/mg and a critical micelle concentration of 13.5 mg/L. FT-IR analysis revealed the presence of basic hydroxyl, carboxyl, ether, amine/amide functional groups, and alkyl aliphatic chains, which is consistent with its lipoglycopeptide nature (60% lipids, 19.6% carbohydrates, and 9% proteins). Gordofactin displayed remarkable stability and retained emulsifying activity across a broad range of temperatures (30 °C to 80 °C) and pH (pH 3–12). Moreover, a significant tolerance of gordofactin emulsifying activity (EA) to a wide range of NaCl concentrations (1 to 100 g/L) was demonstrated. Although with a great loss of EA in the presence of NaCl concentrations above 2.5%, gordofactin could still tolerate up to 100 g/L NaCl, maintaining about 16% of its initial EA for up to 7 days. Furthermore, gordofactin exhibited growth inhibition against both Gram-positive and Gram-negative bacteria, and it demonstrated concentration-dependent free radical scavenging activity for 2,2-diphenyl-1-picrylhydrazyl (IC_50_ ≈ 1471 mg/L). These promising features emphasize the robustness and potential of gordofactin as an eco-friendly BS/BE alternative to conventional surfactants/emulsifiers for different industrial applications.

## 1. Introduction

Biosurfactants/bioemulsifiers (BSs/BEs) can be defined as surface-active biomolecules produced by microorganisms, such as bacteria and fungi, with a wide range of potential applications. They are typically known for their ability to reduce surface tension (surfactant activity) and/or promote the formation of emulsions between two immiscible phases (emulsifying activity). Due to their functional abilities and eco-friendly properties, these compounds have been attracting great interest and are regarded as multifunctional biomolecules of the twenty-first century.

In fact, growing environmental concern combined with stringent regulations culminated in the search for more sustainable alternatives to replace surfactants/emulsifiers of petrochemical origin. Thus, the importance of BSs/BEs on the global market has been growing daily, as they are natural resources with a high added value [1]. Currently, the bio-based surfactants market is projected to grow from USD 19.64 billion in 2024 to USD 25.68 billion by 2032, exhibiting a compound annual growth rate (CAGR) of 3.90% during the forecast period of 2024 to 2032 [2]. In addition, the emulsifiers market will also reach an estimated valuation of USD 17.53 billion by 2027, with this growth registering at a CAGR of 6.90% for the forecast period of 2020 to 2027 [3].

Bio-based surfactants/emulsifiers find applications in a wide range of industries. Their use promotes the development of a circular economy and contributes to the shift towards more sustainable and environmentally friendly practices. Currently, although a considerable amount of work has been carried out to ensure BS/BEs’ economic feasibility, their industrial production is still under development mainly due to the challenges and high costs involved in microbial cultivation and product recovery [4]. Therefore, promising prospects for BSs/BEs to become commercially successful could be through the utilization of renewable and low-cost nutrients (e.g., agricultural/food waste) [5], and/or through coproduction along with other commercially important bioproducts, integrating processes and reducing overall production costs [6].

BSs/BEs are considered advantageous compared to their counterparts of petrochemical origin due to their lower/non-toxicity, biocompatibility, greater biodegradability, better specificity due to their unique structures, good performance at extreme temperatures, pH and salinity, and widespread applicability [7,8]. Indeed, they have been extensively applied in environmental bioremediation, enhanced oil recovery, and industrial emulsification [5,9]; however, with better elucidation of their characteristics, properties, and functions, new, more specific applications have been reported in agrochemicals, food, cosmetics, biomedicals, and therapeutics, namely with antimicrobial, antioxidant, and antiadhesive purposes and many others [5,10,11,12].

The bacterium *Gordonia alkanivorans* strain 1B, isolated from oil-contaminated soil samples by Alves et al. [13], is a well-known, efficient desulfurizing microorganism [14,15,16,17,18,19,20] which is also described as a producer of high-added-value carotenoids [21,22,23]. Recently, it was shown that this bacterium can also produce extracellular biomolecules with both surfactant and emulsifier properties [24,25]; however, there is little information on how these properties are affected by external factors or their potential as antioxidant or antimicrobial compounds. This study classifies the extracellular compounds produced by strain 1B, when cultivated in a chemostat with fructose as a carbon source, as gordofactin and proceeds to determine its in-depth physico-chemical/biochemical characterization, after refined purification, assessing its thermal, pH, and salinity stability and evaluating its antioxidant and antimicrobial properties.

## 2. Material and Methods

### 2.1. Microorganism and Culture Conditions for Biosurfactant Production

The microorganism used in this work was the bacterium *Gordonia alkanivorans* strain 1B, isolated in our laboratory [13] and kept in a culture collection of microorganisms (CCM at LNEG, Portugal, Lisbon). The salt medium used for cultivation of this microorganism in a bioreactor was the minimized culture medium, described by Silva et al. [20], containing 2.2 g/L NH_4_Cl, 1 g/L KH_2_PO_4_, 1 g/L Na_2_HPO_4_·2H_2_O, 0.085 g/L MgCl_2_·6H_2_O, 0.063 g Na_2_SO_4_, and 0.25 mL/L micronutrient solution [14]. This medium was adjusted for a final pH of 7.5 and autoclaved at 121 °C, 1 atm for 15 min. Filter-sterilized fructose solution (50% *w*/*v*) was added to the culture medium to an initial concentration of 20 g/L, as the only carbon source. Thus, the bacterial biosurfactant was produced within a 3.3 L bioreactor (Bioflo III, New Brunswick Inc, Edison, NJ, USA), in continuous culture, with a 0.65–0.70 L working volume, a dilution rate of 0.0675 ± 0.0015 h^−1^, an aeration rate of 2 vvm (volume of air per volume of liquid per minute), and an agitation rate of 425 rpm (revolutions per minute). The working volume was kept constant by using a surface-dipped leveling tube linked to a variable-speed peristaltic pump. The temperature was controlled at 30 °C and pH was controlled by the addition of 2 M NaOH on demand to 7.5. Foam control was carried out by the addition of 0.002% (*v*/*v*) polypropylene glycol (PPG-2000) to the culture medium.

### 2.2. Extraction and Partial Purification of Gordofactin

The culture broth was centrifuged at 13,000× *g* for 40 min at 4 °C. The resulting cell-free supernatant was subjected to ultrafiltration through a tubular membrane (ITT PCI Membranes, Basingstoke, UK) using a maximum pressure of 5 ± 0.5 bar and a nominal molecular weight cut-off (MWCO) of 15 kDa. A sample of the recovered retentate (gordofactin concentrate—GC-15) was extracted with chloroform–methanol (2:1) at room temperature. In a separatory funnel, the chloroform–methanol (2:1) was mixed with the GC-15 in a 1:1 ratio, shaken vigorously for 10 min, and then left stationary for phase separation. After stabilization, two distinct phases were observed, an upper aqueous–methanolic phase containing a white precipitate and a lower orange phase containing carotenoids originally present in the culture broth. The chloroform lower phase with the extracted carotenoids was collected and another equal volume of chloroform–methanol (2:1) was added. The extraction process was repeated until there was no color in the chloroform layer. After extraction, the aqueous–methanolic fraction containing the white precipitate was collected, homogenized, and analyzed (EG—extracted gordofactin, i.e., the partially purified gordofactin sample). The EG sample was further lyophilized (BK-FD12P (−80 °C) Vacuum Lyophilizer, Biobase Biodustry Co., Ltd., Jinan, China), resulting in a powder that was designated as lyophilized extracted gordofactin (LEG), henceforth used in the characterization tests.

For further purification, a small amount of LEG (lyophilized EG) was redissolved in ultrapure water (i.e., Milli-Q water; PURIST® System, RephiLe Bioscience Ltd., Lisbon, Portugal) and subjected to ultrafiltration through a 1000 kDa centricon filter (Vivaspin^®^ 20 PES MWCO 1000 kDa, Sartorius, Gottingen, Germany). This second retentate (gordofactin concentrate—GC-1000) was lyophilized (LGC-1000—lyophilized GC-1000) and its emulsifying activity and critical micelle concentration were evaluated for comparison.

### 2.3. Emulsifying Activity Determination

The emulsifying activity (EA) was carried out according to the method established by Tavares et al. [24]. Aliquots of the gordofactin solutions, from samples before and after purification (namely: direct cell-free supernatant, ultrafiltered sample, and extracted sample), were diluted in ultrapure water, up to 1 mL aqueous fraction, in 4 mL screw cap glass tubes (10 × 75 mm, ND10 caps with a PTFE septum), and then 1 mL of n-heptane was added and mixed via vortexing at high speed for 2 min. Then, the tubes were left to rest vertically for 10 min for emulsion detection. A set of emulsification tests with increasing volumes of each gordofactin sample solution was carried out until 100% emulsion was obtained in the organic phase. According to Tavares et al. [24], one emulsification unit (1 U) consists of the minimum volume of product (Vol_min_ of gordofactin sample) needed to form and maintain 100% emulsion in 1 mL of n-heptane. Thus, the corresponding EA value is presented in U/mL and calculated as: EA (product) = 1 U/Vol_min_ (mL). Furthermore, based on the concentration of gordofactin in each tested sample, the corresponding specific emulsifying activity (SEA), in U/g, was also estimated.

### 2.4. Suspended Solids Determination

Samples were added to pre-dried (105 °C in oven, overnight) and pre-weighed Eppendorf tubes and then dried at 105 °C overnight in a convection oven. Thereafter, the Eppendorf tubes with samples were cooled in a desiccator, and then the final dry weight (g/L) was determined using an analytical balance (GR-202, A&D Company, Tokyo, Japan).

### 2.5. Gordofactin Chemical and Biochemical Composition

Chemical and biochemical characterization was carried out using the more purified sample of gordofactin obtained in this study (i.e., the LEG sample). CHNS elemental analysis was performed following ISO 16967:2015 [25]. The total carbohydrate content was estimated using the phenol–sulfuric acid method [26]. Protein concentration was estimated by using the modified Lowry procedure at room temperature [27]. For lipid content estimation, 50 mg of LEG was extracted with 500 µL of chloroform:methanol (2:1 *v*/*v*). The extraction was repeated three times, and the combined organic extracts were evaporated at 50 °C. The lipid content was determined gravimetrically [28]. All chemical/biochemical composition tests were performed in triplicate.

### 2.6. FT-IR Spectroscopy Detection

The functional groups present in the gordofactin lyophilized samples, from the two stages of partial purification (GC-15 sample and two distinct batches of the EG sample), were analyzed using a Fourier transform infrared (FT-IR) spectrophotometer (Perkin Elmer Spectrum BX, Waltham, MA, USA) equipped with Spectrum software v5.3.1 for data analysis, by adopting potassium bromide (KBr) pellet method, in the spectral range of 4000–400 cm^−1^.

### 2.7. Surface Tension and Critical Micelle Concentration Determination

The surface tension (ST) of various gordofactin samples, at different stages of purification, was determined using a KRÜSS K12 MK6 tensiometer at 25 °C ± 1, as described by Silva et al. [25]. For each lyophilized sample, a set of samples of increasing concentration were prepared in ultrapure water (GC-15: 1.37 to 431.80 mg/L; EG: 4.72 to 471.74 mg/L; GC-1000: 3.71 to 593.75 mg/L]). All ST measurements were performed using the Du Noüy ring method and the ST of ultrapure water (72 mN/m) was used to calibrate the tensiometer. For each gordofactin tested sample, the corresponding set of the progressively greater concentrations was measured 10 times, after 2 min of stabilizing, and the ST average values obtained (mM/m) were plotted and used to calculate the respective critical micelle concentration (CMC), as the concentration in which a sudden change in ST behavior is noted. CMC values obtained for Tween 80 and Sodium Dodecyl Sulfate (SDS), by Silva et al. [25], were used as global reference positive controls.

### 2.8. Thermal, pH, and Salinity Stability Tests

For the stability tests, the LEG sample was used to prepare the aqueous solutions with known initial emulsifying activity (EA ≈ 22 U/mL). The thermal stability of gordofactin emulsifying properties was evaluated by determining the EA, according to Tavares et al. [24] as described above (Section 2.3), of gordofactin solutions (LEG dissolved in ultrapure Milli-Q water) incubated at 30, 50, 70, 80, and 90 °C for up to 8 h. To evaluate the pH effect/stability, different gordofactin solutions were prepared by dissolving aliquots of LEG in ultrapure water adjusted at a pH range of 2 to 12. Ultrapure water, presenting a pH of 6.1, was adjusted to the different pH tested using HCl 1M or NaOH 1M solutions, for acidic or alkaline pH respectively. The effect of pH on gordofactin EA was also evaluated by determining the EA after 1 h of dissolution/exposure and after 7 days of storage at 4 °C. Furthermore, to evaluate saline effect/stability, different solutions were prepared by dissolving LEG on ultrapure aqueous sodium chloride solutions ranging from 1 g/L to 100 g/L. The EA of gordofactin in different saline solutions was evaluated after 1 h of exposure and after 7 days of storage at 4 °C. The results are presented in %EA in comparison with the EA observed in the best condition (for each parameter tested, gordofactin solution with the highest EA was considered as 100% EA).

### 2.9. Determination of Antimicrobial Activity

The antimicrobial activity of the LEG sample was studied against two Gram-positive bacteria (*Staphylococcus aureus* and *Bacillus subtilis*) and two Gram-negative bacteria (*Pseudomonas putida* and *Escherichia coli*) and two fungi (*Candida albicans* and *Aspergillus brasiliensis*) kept in a culture collection of microorganisms (CCM at LNEG, Portugal, Lisbon). Flat-bottom 96-well sterile microtiter plates with lids were used in the inhibition growth assays. The different stock cultures (25 mL) were prepared by inoculating 2% of overnight grown cultures of bacteria and fungi in new culture medium (nutrient broth (NB) and yeast malt broth (YMB), respectively). Firstly, aliquots of 100 µL of these stock cultures were transferred to the microplate wells (final concentration ≈ 10^6^ cells/mL), with each microorganism in a row with one row of intervals to avoid cross-contamination. Then, aliquots of 100 µL of different UV light-sterilized aqueous solutions of gordofactin were added to the wells (in triplicate) to obtain final concentrations of 2.5, 5, 10 and 15 g/L. As growth controls, 100 µL of the culture medium inoculated with each of the microorganisms was mixed with 100 µL of distilled water (absence of gordofactin). Negative controls were prepared using 100 µL of uninoculated medium and 100 µL of distilled water (absence of both the test microorganism and gordofactin). The microplate was placed in a microplate reader (Multiskan, Thermo Fisher Scientific Inc., Waltham, MA, USA) and incubated at 30 °C for 24 h with constant agitation. The optical density at 600 nm (OD_600_) was measured prior to incubation (T_0_) and every 60 min (T_i_) until the end of the incubation time. The effect of gordofactin on the growth/survival of each test strain was evaluated by plotting the growth curves, with OD_600_ = OD_600_ (T_i_) − OD_600_ (T_0_). All of the values obtained were an average of at least triplicates.

### 2.10. Determination of Antioxidant Activity

The antioxidant activity of the more purified sample of gordofactin (LEG) was estimated using DPPH (2,2-diphenyl-1-picrylhydrazyl) free radical scavenging activity (% RSA) according to Prieto’s DPPH Microplate Protocol [29], with slight modification. Stock solutions of the gordofactin (6.6 g/L) and L(+)-ascorbic acid from Merck (1 g/L), as a positive control (standard antioxidant), were prepared using ultrapure water (Milli-Q water), and then they were serially diluted in a 96-well microplate to form several 100 µL double dilutions in each well. The negative control (control blank) consisted of a blank with 100 µL of Milli-Q water. Then, 100 µL of the DPPH 0.2 mM solution in absolute ethanol was added to the control blank and the serial concentrations of gordofactin (50–6600 mg/L) and L-ascorbic acid (0.3–62.5 mg/L). To the blank samples, 100 µL of absolute ethanol was added instead. The microplate was covered with the lid to minimize evaporation and wrapped in foil, and, after being gently shaken for 2 min for mixture homogenization, it was left in the dark at room temperature for 30 min, after which the OD was measured at 517 nm in a microplate reader (Multiskan, Thermo Fisher Scientific Inc., Waltham, MA, USA). All conditions were tested at least in triplicate. The percentage of DPPH scavenging was determined as follows: DPPH RSA (%) = [1 − (OD.Sp − OD.SpBk)/OD.Bk] × 100, where OD.Sp is the optical density of the sample and OD.SpBk is the optical density of the blank with the sample and OD.Bk is the optical density of the blank without the sample (control blank). Then, for each tested sample, the % DPPH RSA was plotted against the concentration (mg/L or g/L), and the inhibitory concentration (IC_50_—the concentration where 50% inhibition of the DPPH radical is obtained) was estimated.

## 3. Results and Discussion

### 3.1. Gordofactin Extraction, Partial Purification Versus EA and CMC

*G. alkanivorans* strain 1B was cultivated in a chemostat using a minimal medium with fructose as the sole carbon source, as described above (Section 2.1), without the addition of any hydrophobic inducer. Under these conditions, both pigmented cells (desulfurizing biocatalysts, 8.42 ± 0.5 g/L) and the gordofactin (extracellular BSs/BEs) were continuously produced at a production rate of 1.2 L per day; however, the focus of this study was only the gordofactin characterization.

Therefore, the cell-free supernatant containing gordofactin was subjected to different purification steps. Indeed, classic methods of biosurfactant separation from cell-free culture broth are intricate processes that require significant quantities of organic solvents and generate substantial solvent waste, rendering them nearly infeasible for medium or large-scale production. Ultrafiltration technology harnesses the propensity of biosurfactants to form micelles and aggregates at concentrations above the critical micelle concentration (CMC) to enable their retention by relatively high molecular weight cutoff membranes, while impurities such as salts, free amino acids and peptides, and small proteins can be readily eliminated along with water removal [30,31].

**Figure 1:**
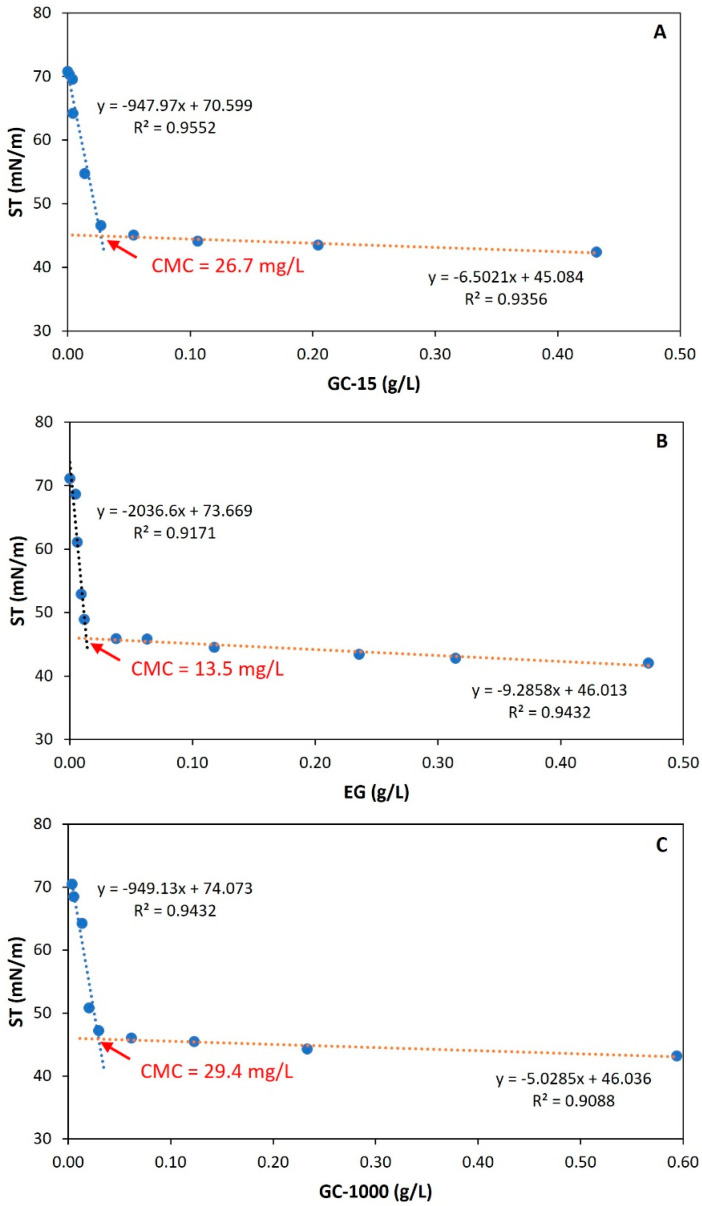
Surface tension (ST) and critical micelle concentration (CMC) of gordofactin samples at different purification stages: (**A**) GC-15—gordofactin concentrate from ultrafiltration by the 15 kDa MWCO membrane; (**B**) EG—extracted gordofactin (solvent extraction); (**C**) GC-1000—gordofactin concentrate from ultrafiltration by the 1000 kDa MWCO membrane. Data points represent averages of ten measurements of each replicate (0.06% < SD < 2.63%, SD—standard deviation).

In Table 1 and Figure 1, an initial characterization of gordofactin is presented based on its emulsification and surfactant properties [i.e., EA (U/mL) and CMC (mg/L)], throughout the different purification steps, i.e., from raw supernatant (cell-free supernatant) up to ultrafiltered concentrates (GC-15; GC-1000) or the extracted sample (EG).

In the first separation step, the cell-free supernatant with gordofactin underwent ultrafiltration through a membrane with a molecular weight cutoff of 15 kDa leading to the concentration of the suspended components ranging from 8.69 g/L to 15.51 g/L (Table 1). Simultaneously, the volumetric emulsifying activity (EA) in this gordofactin concentrate (GC-15) increased from 40 U/mL to a remarkable 144 U/mL, while the permeate contained only an EA of less than 1 U/mL. Therefore, this ultrafiltration step seems to have effectively removed water, soluble salts, and a range of components smaller than the 15 kDa molecular weight cutoff, which had no substantial impact on the gordofactin emulsifying activity. This GC-15 presented an estimated specific emulsifying activity (SEA) of 9.3 U/mg. The further lyophilization of GC-15 yielded an orangish powder with an SEA of 13.5 U/mg, which was designated as the lyophilized gordofactin concentrate (LGC-15).

In the next purification step, the GC-15 was partitioned via liquid–liquid extraction, resulting in the extraction of pigments and free lipids into the chloroform fraction. The aqua-methanolic fraction, containing virtually all of the initial emulsifying activity (99.5%), retained a whitish precipitate designated as extracted gordofactin (EG). This precipitate contained 7.36 ± 0.12 g/L of suspended solids and demonstrated an SEA of 11.3 U/mg. Upon lyophilization, the EG yielded a whitish powder, maintaining a specific emulsifying activity of 9.5 ± 0.2 U/mg (Table 1), indicating a slight decrease from the initial value.

During attempts to further purify the gordofactin components, the LEG sample was resuspended and subjected to a stricter ultrafiltration procedure using 300 and 1000 kDa MWCO membrane filters (Vivaspin 20 PES filters). Ultrafiltration using the 300 kDa filter resulted in the maintenance of its EA, while ultrafiltration through the 1000 kDa filter resulted in a marginal increase in SEA to 10.96 U/mg (Table 1), which appears to be attributed to the removal of components with molecular weights below 1000 kDa but above 300 kDa.

However, to decide which was the best gordofactin sample, i.e., the more purified form, for further detailed characterization, the surfactant activity was very relevant. In fact, the effectiveness of surfactant compounds, including both biosurfactants and synthetic surfactants, can be evaluated by their ability to reduce surface tension, and the CMC is a crucial parameter influencing the primary characteristics of these surfactants. Therefore, in this study, the CMC was evaluated during the different steps of gordofactin purification (Figure 1) for comparison and for the selection of the more purified sample exhibiting the highest surfactant activity for further detailed characterization. The CMC values were determined as the concentrations at which a sudden change in the downward trend of surface tension occurs, followed by much smaller decreases in these values despite the increase in the surfactant’s concentration. As can be seen in Figure 1A, after the first step of purification, the LGC-15 sample presented a CMC of about 26.7 mg/L with a corresponding surface tension of 45.29 mN/m. The LEG, resulting from the sequential solvent extraction purification stage, had its CMC improved to about 13.5 mg/L (Figure 1B) with the corresponding surface tension of 45.97 mN/m. However, the GC-1000, i.e., the LEG after ultrafiltration through the 1000 kDa MWCO membrane to exclude compounds below 1000 kDa, presented a significant increase in its CMC value, from 13.5 mg/L to 29.4 mg/L, with a corresponding surface tension of 46.17 mN/m. This increase in CMC indicates the loss of surfactant activity of gordofactin associated with the extraction of important components. Therefore, the most adequate purified gordofactin sample for further detailed characterization was the LEG.

In addition, the CMC value obtained for the LEG (13.5 mg/L) is comparable or significantly lower to those obtained for reference surfactants with high purity (e.g., Tween 80: CMC = 6.4 mg/L; SDS: CMC = 1840 mg/L) [25]. As mentioned by Otzen [32], the lower the CMC, the better the biosurfactants self-associate to form micelles which improve their efficiency in various applications, such as solubilizing hydrophobic compounds.

Moreover, independently of the purification step used (ultrafiltration or solvent extraction), all partially purified gordofactin samples obtained met the benchmark value of ≥20 mN/m in water surface tension reduction, a critical indicator of biosurfactant efficacy. This finding aligns with those previously reported by Silva et al. [25], who employed a distinct extraction method to obtain comparable surfactant properties. It is also noteworthy that the surface tensions obtained for all tested gordofactin samples (45.29, 45.97, and 46.17 mN/m for GC-15, EG, and GC-1000, respectively) are relatively close to those observed by Silva et al. [25] for Tween 80 (44.40 mN/m) and SDS (37.92 mN/m), suggesting that the surface activity of the gordofactin is also comparable to that observed for these reference synthetic surfactants, despite being only partially purified. In the case of the SDS, the concentration required to achieve this surface tension was at least one order of magnitude higher than that of gordofactin, indicating its potential to compete with synthetic surfactants.

Although reports on the characterization of *G. alkanivorans* biosurfactants are scarce, the ability of *Gordonia* species to produce biosurfactants with high surface properties has been widely documented [33,34,35,36,37,38]. This likely stems from their need to thrive in environments often contaminated by hydrophobic hydrocarbons. The biosurfactants they produce enhance access to these compounds, which serve as carbon sources. This ability provides a competitive advantage in these extreme and resource-scarce habitats, holding great potential for biotechnological applications such as bioremediation of oil spills.

### 3.2. Gordofactin Structural Characterization

#### 3.2.1. Chemical/Biochemical Composition

The unique biochemical composition of biosurfactants/bioemulsifiers (BSs/BEs), linked to their microbial origin and functional properties, grants them advantages over their synthetic counterparts, particularly greater biodegradability and lower toxicity [39,40]. Despite their diverse structures posing analytical challenges due to the impact of production conditions and purification processes on their molecular characteristics and functionalities, thorough macromolecular and biochemical characterization remain paramount for their successful practical application.

The elemental content (CHNS) of the more purified gordofactin (LEG sample), produced by *G. alkanivorans* strain 1B, was determined, and the findings demonstrated that it contains 30.95% carbon, 3.73% hydrogen, and 2.43% nitrogen. The average sulfur content was less than 0.3%. Based on this result, the high carbon content indicates that carbon-containing organic compounds are the major component. The hydrogen content suggests the presence of hydrocarbon structures, and the nitrogen (2.43%) and sulfur (<0.3%) content indicate the presence of nitrogen-containing functional groups (amine or amide) and sulfur-containing amino acids. This aligns with the hypothesis that gordofactin is likely composed of a complex mixture of the main biological macromolecules: lipids, carbohydrates, and proteins.

Further detailed biochemical characterization showed that the LEG sample is primarily composed of lipids (60%), followed by carbohydrates (19.63%) and proteins (9.0%). This suggests that the gordofactin produced by strain 1B is consistent with a lipoglycopeptide or a mixture of lipopeptides, glycopeptides, and/or glycolipids, as it contains significant amounts of all three major classes of macromolecules (lipids, carbohydrates, and proteins). The lipoglycopeptide nature of the BS/BE produced by strain 1B has already been identified by Silva et al. [25]. The difference observed in biochemical composition is directly related to the lower purification degree of the gordofactin sample and/or to the extraction method used previously.

The biosurfactant’s structural nature can be inferred from the biochemical components that make up the compound or molecule. However, its classification also depends on its microbial origins and the diversity of its biological and chemical activity. They can be low-molecular-weight biosurfactants, such as glycolipids, phospholipids, and lipopeptides, and high-molecular-weight biosurfactants/bioemulsifiers, including polysaccharides, proteins, lipopolysaccharides, lipoproteins, or complex mixtures of these biopolymers [41,42].

Bacteria belonging to the genus *Gordonia* have been reported to produce various types of biosurfactants, including extracellular and/or cell-bound forms. Several studies have identified these biosurfactants as trehalose lipids, lipopeptides, glycolipids, and polymeric glycolipids [33,34,35,36,38]. Laorrattanasak et al. [37] reported a biosurfactant produced from *G. westfalica* GY40 containing 37% lipid, 8% protein, and 32% sugar, indicating that it is a potential glycolipid. In this study, it is shown that the gordofactin produced by *G. alkanivorans* strain 1B, exhibiting both emulsification and surfactant properties, is considered a lipoglycopeptide BS/BE.

#### 3.2.2. Functional Group Detection via FTIR Spectroscopy

**Figure 2:**
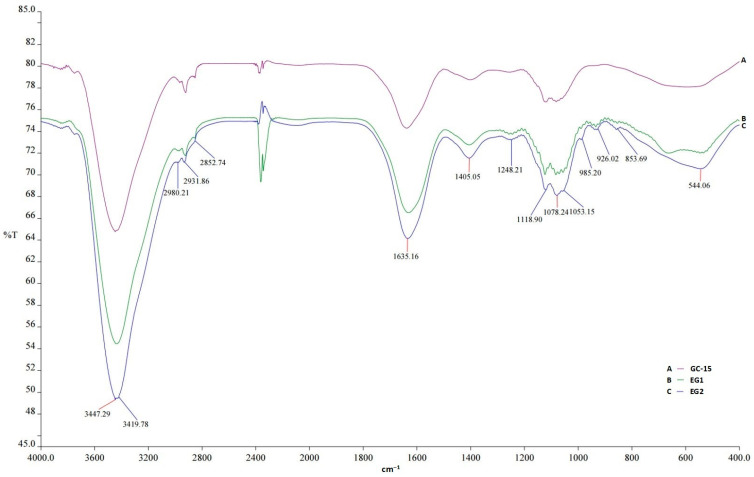
The FT-IR transmittance spectra of partially purified gordofactin samples. A: GC-15—gordofactin concentrate from ultrafiltration by the 15 kDa MWCO membrane; B: EG1—extracted gordofactin—batch 1; C: EG2—extracted gordofactin—batch 2.

Fourier-transform infrared spectroscopy (FT-IR) is a particularly useful technique for identifying the types of functional groups and the chemical bonds present in a purified fraction of a novel BS/BE sample, contributing to the elucidation of its chemical nature. In this context, the lyophilized GC-15 (from the ultrafiltration step) and the lyophilized EG samples (EG1, EG2—extracted gordofactin samples from two different batches, i.e., replicates) were subjected to FT-IR analysis, and the corresponding spectra are illustrated in Figure 2.

As can be observed in this figure, the three spectra revealed highly similar profiles, which seem to demonstrate that the fine components of gordofactin are maintained after both purification steps (i.e., ultrafiltration + solvent extraction). Spectral analysis showed strong broad absorption peaks centered around the 3447 cm^−1^ region, corresponding to the characteristic O–H stretching vibration (free hydroxyl groups) found in a wide variety of sugar moieties, amino acids, and fatty acids and the N–H stretching vibration present in the peptide moiety [43,44,45,46,47]. In fact, a protein peptide bond is an amide group. The absorption spectrum bands assigned as amide I (1600–1800 cm^−1^), amide II (1470–1570 cm^−1^), amide III (1250–1350 cm^−1^), and amide A (3300–3500 cm^−1^) are peaks of infrared characterization of amide compounds [48]. Therefore, in gordofactin IR spectra, besides amide A bands (near 3500 cm^−1^), other protein-related bands were also observed around 1635 cm^−1^ (amide I), assigned to the C=O stretching vibration of the amide group (C=O–NH), which represented the conjugation between the amine group of the amino acid with the carboxylic group of the fatty acid, and around 544 cm^−1^, assigned to N–H oscillation [48]. The peaks centered around 2980, 2932, and 2853 cm^−1^ were assigned to C–H stretches characteristic of alkyl groups (–CH, –CH_2_, and –CH_3_) of the long aliphatic C–C chain, which seems to be further evidenced by the bending vibration peaks at around 1405 cm^−1^ [44,46,49]. These C–H stretch peaks are characteristic of lipids (fatty acids), proteins [50], and carbohydrates/cellular polysaccharides [43,44,46]. Usually, in carboxyl acid-based compounds (e.g., fatty acids, amino acids), absorption peaks due the C–O stretch appear in the region 1210–1320 cm^−1^ (e.g., peaks around 1248 cm^−1^—Figure 2), and those of the O–H bend appear in the regions 1395–1440 cm^−1^ and 910–950 cm^−1^ [51], although the 1395–1440 cm^−1^ O–H bending band may not be distinguishable from C–H bending bands in the same region (e.g., peaks observed at around 1405 cm^−1^—Figure 2) [44]. In addition, the stronger/weaker absorption peaks observed in the region 1200–900 cm^−1^ (i.e., 1118, 1078, 1053, 985, and 926 cm^−1^—Figure 2) are mainly dominated by a sequence of bands usually assigned to C–O, C–O–C, C–C and C–O–P stretching vibrations, from functional groups prominent in carbohydrates/polysaccharides [44,46,50].

Thus, the FT-IR analysis revealed the presence of basic hydroxyl, carboxyl, ether, amine/amide functional groups and alkyl aliphatic chains, which is consistent with the lipoglycopeptide nature observed for gordofactin. The different FTIR profiles clearly demonstrate a different composition when compared to previously described biosurfactants produced by *Gordonia* sp. IITR100, which lacked the amine/amide group, indicating that these are different compounds, despite some similarities [38]. Although the FT-IR results agreed with those from the biochemical composition analysis, the detailed structure of the gordofactin requires further analysis. However, since gordofactin is only soluble in water and insoluble in most usual organic solvents, it was not possible to perform NMR nor GC-MS spectral analyses for structural elucidation.

### 3.3. Thermal, pH, and Salinity Stability

The applicability of BSs/BEs in a wide range of industrial applications is predominantly influenced by their robustness and effectiveness under varying environmental or processual conditions, such as temperature, salinity, and pH. Therefore, their resistance to a wide range of these conditions gives them an advantage in many applications, such as the food processing industries, pharmaceuticals, cosmetics, and bioremediation in adverse natural environments.

#### 3.3.1. Effect of Temperature

**Figure 3:**
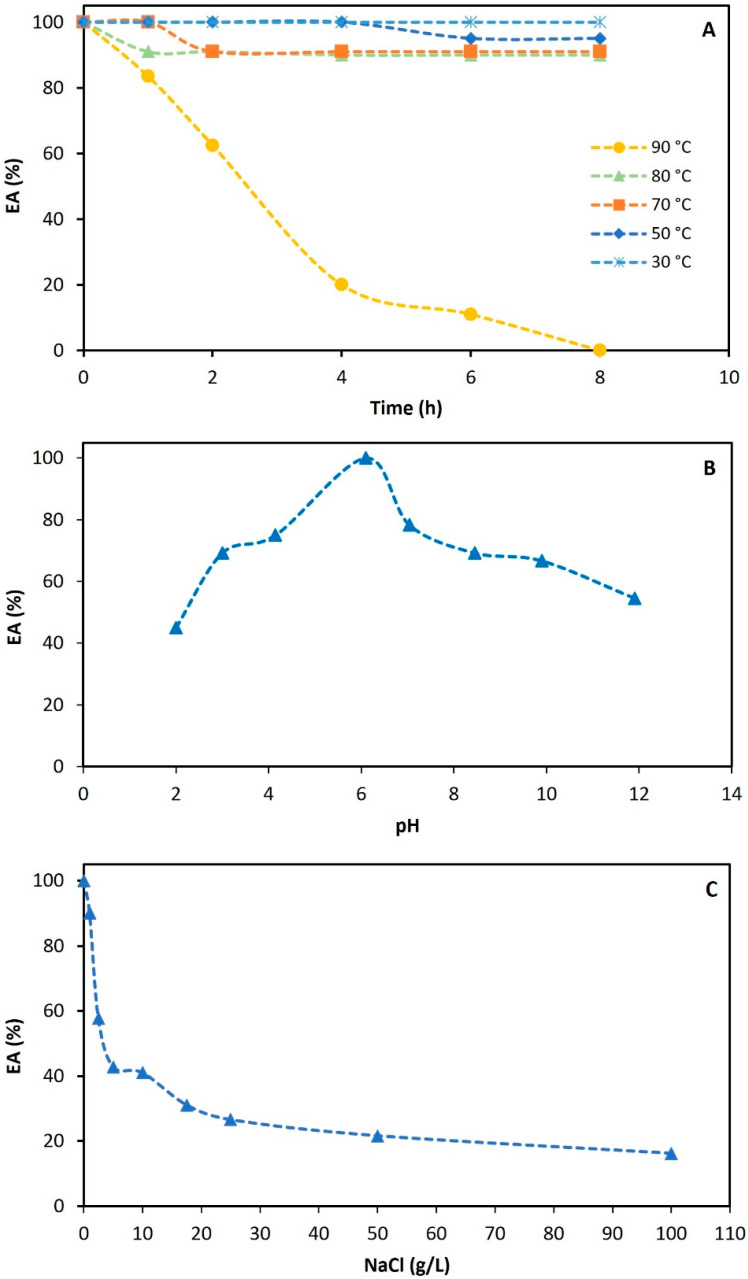
Influence of temperature (**A**), pH (**B**), and salinity conditions (**C**) on the emulsifying activity (EA) of extracted gordofactin. For each factor, the results are presented in percentage of EA in relation to the maximum EA, considered 100%.

Thermostable BSs/BEs hold particular importance as they can retain their functional properties at higher temperatures, broadening the range of industrial application fields and offering numerous advantages over conventional surfactants/emulsifiers. In this study, the stability of extracted gordofactin (LEG sample) was tested over a wide range of temperatures, from 30 °C to 100 °C, with the effect on its emulsifying activity (EA) being evaluated. Thus, the EA of the EG solutions (EA _initial_ ≈ 20 U/mL) subjected to different heat treatments, in a water bath, was determined over the incubation time (up to 8 h), and the results of temperature influence on EA, in percentage (i.e., % relative to the maximum EA at 0 h, considered 100%), are presented in Figure 3A. As can be observed, the EA was virtually stable over the temperatures ranging from 30 °C to 80 °C. The gordofactin solution exposed to 30 °C kept the EA unchanged (with 20 U/mL) for at least 3 months. When kept at 50 °C, there was a slight drop in the EA after 6 h of exposure (Figure 3A), but after that, the EA remained unchanged, at ≈95%, for at least two weeks. Similarly, when the gordofactin solutions were exposed to temperatures of 70 °C and 80 °C, there was a drop of up to about 10% in EA in the first two hours, after which the EA remained stable at ≈90% up to 8 h of exposure (Figure 3A). Conversely, when a gordofactin solution was incubated at 90 °C a substantial decrease was observed in its EA throughout the 8 h of incubation. Notably, during the first hour at 90 °C, the gordofactin still retained over 80% of its initial EA. In fact, the decrease in EA followed a linear trend during the initial 4 h of exposure, leading to a loss of ≈80% of the original emulsifying ability. After 8 h of incubation at 90 °C, the gordofactin solution irreversibly lost its emulsifying ability. This EA loss due to long exposure to high temperatures can be explained by denaturation of the biosurfactant/bioemulsifier protein fraction [52], as seen with other microbial biosurfactants [53].

These promising results of high-stability EA within a wide range of temperatures (at least up to 80 °C) for gordofactin significantly surpass those obtained with the biosurfactant produced by *Gordonia* sp. IITR100, which was only stable between 4 and 30 °C [38], and highlight its potential application in processes where heating to achieve sterility is indispensable or in scenarios where heat is either crucial or directly involved in the desired reactions.

#### 3.3.2. Effect of pH

Figure 3B demonstrates the impact of water pH (ranging from 2 to 12) on the EA of the extracted gordofactin (LEG sample), after 1 h of its dissolution. Gordofactin revealed remarkable stability across a broad pH range, maintaining its EA above 66.7%, in comparison with the maximum EA value observed (i.e., LEG dissolved in ultrapure water, exhibiting pH = 6.10, presented an EA = 22.22 U/mL ⇒ 100% EA), for pHs between 3 and 12. This aligns with similar findings for various microbial biosurfactants, namely glycolipids and lipopeptides like rhamnolipids [54], trehalolipids [55], sophorolipids [56], and surfactin [57], known for their wide pH stability. Compared to the biosurfactant produced by *Gordonia* sp. IITR100, though the pH profile is similar, there is a clear difference in optimal pH, which tends more towards neutrality [38].

However, significant activity reduction may occur at both pH extremes: acidic and alkaline conditions. This effect likely stems from structural modifications induced by harsh pH exposure. In extreme acidic conditions, coalescence of bubbles into larger structures may rapidly destabilize the emulsion, suggesting a loss of emulsifying properties. This aligns with reported observations for microbial biosurfactants [54,58,59,60] and can be attributed to changes in surface charge caused by the protonation of biosurfactant functional groups, leading to electrostatic repulsion, coalescence, and emulsion breakdown. Conversely, at very high pH (>10), deprotonation of functional groups (e.g., free carboxylic acids in glycolipids/lipopeptides) within interfacial biosurfactant molecules may trigger repulsive interactions, causing them to distance themselves. Ultimately, this can lead to the breakdown of emulsion droplets, as reported by Baccile et al. [61]. Furthermore, extreme pH values (both highly acidic and highly alkaline) can induce denaturation of peptide fractions and render various functional groups susceptible to hydrolysis. These combined processes can also further contribute to a loss of emulsifying properties.

In fact, when the LEG sample was dissolved in ultrapure water adjusted at pH 2, its EA was greatly reduced to 45% (relative % in comparison with the highest EA value observed at pH 6.10). In the same way, the EA was also considerably reduced when LEG was dissolved in distilled water adjusted to pH = 12 (54.5% EA).

In addition, this study found that storing the different samples of gordofactin (LEG solutions) dissolved in ultrapure water adjusted under a range of pH between 3 and 12, under refrigerated conditions (4 °C) up to 7 days, did not significantly impact its EA. Indeed, each EA remained ≥81.3%, relative to the respective initial value observed, across a pH range of 3 to 12. However, when dissolved in water with pH = 2, a sharp decline in gordofactin EA was observed, namely from 100% to 45% within 1 h after dissolution, from 45% to 16.4% within 48 h and to 9.8% within 7 days, demonstrating a pronounced time-dependent effect. Nevertheless, this also highlights the great resistance of gordofactin to this extreme acidic pH since it still maintained some emulsifying activity after 7 days at pH 2 (i.e., EA = 2.17 U/mL corresponding to 9.8% EA in comparison to the maximum EA observed).

#### 3.3.3. Effect of NaCl

For some operations, BSs/BEs must be stable and effective across a wide range of salt concentrations to ensure their applicability. In the present study, the tolerance of the extracted gordofactin to increasing NaCl concentrations was evaluated by dissolving the LEG sample under a range of saline concentrations between 1 and 100 g/L (i.e., 0.1% to 10% NaCl aqueous solutions). The EA was measured in these different solutions, after 1 h of exposure. The results obtained are calculated in relation to the sample with the highest EA (LEG solution without NaCl ⇒ 100% EA, corresponding to 22.2 U/mL). As shown in Figure 3C, gordofactin decreased its EA with the increase in the NaCl concentration tested. A sharp decrease, to about 42.8% EA, was observed in the range from 1 g/L to 5 g/L of NaCl, and then a slower decrease, from 41% to 16.2%, was observed in the range between 10 g/L and 100 g/L of NaCl. However, after 7 days of being stored at 4 °C, the eight gordofactin saline solutions maintained the respective % EA without significant changes, which indicates that the loss of emulsifying activity observed is intrinsic to the dissolution process in each saline solution concentration.

These results are aligned with those obtained with other *Gordonia* [38], as well as numerous studies that have demonstrated the high tolerance of microbial biosurfactants to saline concentrations, as reviewed by Kumar et al. [62], and Sarubbo et al. [63]. This highlights their distinct advantage over synthetic surfactants, which often exhibit susceptibility and lose activity at ≥2% NaCl concentrations [1].

Herein, although gordofactin seems to be sensitive to NaCl concentration (Figure 3C), with a great loss of EA (>73%) in the presence of NaCl concentrations > 25 g/L (2.5%), it can still tolerate up to 100 g/L NaCl with some EA (16.2% of initial EA corresponding to 3.6 U/mL) for at least 7 days. This highlights the robustness of gordofactin.

### 3.4. Antimicrobial Activity

Considering the putative identification of gordofactin (EG/LEG) as a lipoglycopeptide, based on its biochemical composition and FT-IR analysis, it is important to note that this class of macromolecules is well known for its broad antimicrobial activity, primarily targeting the Gram-positive bacterial cell wall and preventing its formation through inhibition of transpeptidation, ultimately leading to bacterial death [64].

**Figure 4:**
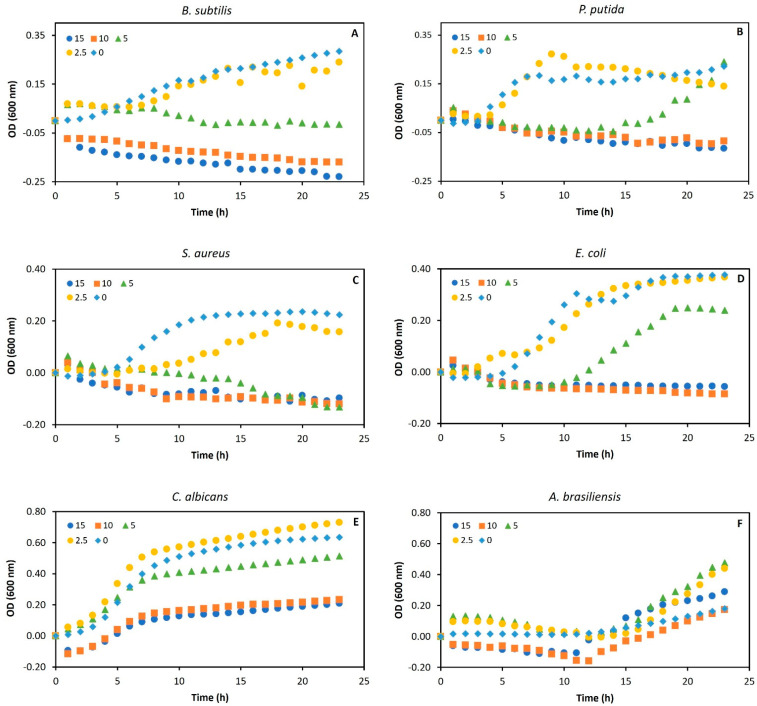
Growth profiles of six microbial strains in the presence of different concentrations (0, 2.5, 5, 10, and 15 g/L) of extracted gordofactin (EG) produced by *G. alkanivorans* strain 1B. Gram-positive bacteria: *B. subtilis* (**A**) and *S. aureus* (**C**), Gram-negative bacteria: *E. coli* (**B**) and *P. putida* (**D**), and fungal strains: *C. albicans* (**E**) and *A. brasiliensis* (**F**). Each graphical point represents the average of at least 3 experimental measurements (SD ≤ 5.0 ± 0.5%). Note: For each time (t_i_), OD600 = OD600 (t_i_) − OD600 (t_0_).

In this context, the antimicrobial activity potential of gordofactin (EG) was studied by testing its effect on the growth of six microbial strains, at concentrations ranging from 0 to 15 g/L. These strains included two Gram-positive bacteria (*B. subtilis* and *S. aureus*), two Gram-negative bacteria (*E. coli* and *P. putida*), and two fungal strains (*C. albicans* and *A. brasiliensis*). The growth profiles obtained for the range of concentrations tested, outlined in Figure 4A–F, demonstrate that the extracted gordofactin exhibited antimicrobial activity against all bacterial strains tested but no significant inhibitory activity against the fungal strains. For all microbial tests, no growth was observed in the respective negative control, indicating no contamination during the time course of each growth assay.

For Gram-positive bacteria, concentrations greater than 5 g/L of gordofactin were sufficient to inhibit growth (Figure 4A,C). However, Gram-negative bacteria required higher concentrations (10 g/L) for complete inhibition (Figure 4B,D). At 5 g/L, these bacteria exhibited an extended lag phase but eventually overcame the inhibitory effect and grew. On the contrary, both fungal strains were able to grow in the presence of all tested concentrations (Figure 4E,F), and, in some cases, they grew to a higher density than in the control without gordofactin. For *A. brasiliensis*, all growth profiles presented a great lag phase (≈12 h), from 0 to 15 g/L gordofactin, potentially indicating some nutrient deficiency in the general culture medium used to grow this strain (YMB). However, after the lag phase, this fungus grew in all gordofactin concentrations tested.

These results can be attributed to the specific mechanism of action of BSs/BEs on microorganisms. Unlike many bacterial species, where BSs/BEs often effectively target and disrupt key components of the cell membrane, fungal cell walls provide enhanced rigidity and protection against such disruption. Thus, the reduced sensitivity to gordofactin by both tested fungal strains may be linked to the distinctive biological and chemical properties of their cells, as well as to the inherent nature and specificity of gordofactin. This antifungal resistance is further supported by findings such as those reported by Jasin et al. (2016) [65], which demonstrate the limited effectiveness of some highly potent biosurfactants, including surfactin, against pathogenic fungi of medical importance.

Biosurfactants with high surface-active properties often exhibit some degree of antimicrobial activity. Lipopeptides, glycolipids, and lipoglycopeptides, known for their strong emulsifying and surface-active properties, have been extensively investigated for their potential as antibiotics [66]. This interest is particularly high given the progressive increase in microbial resistance to conventional antibiotics.

The biosurfactants produced by the genus *Gordonia* are reported as glycolipids, lipopeptides, polymeric glycolipids and possible lipoglycopeptides [33,34,35,36,37]. The exhibition of antimicrobial properties may be beneficial in extreme environments, where these microorganisms are found, characterized by intense competition for scarce resources. However, even if these BSs/BEs with good surface activity lack antimicrobial properties, they may be promising for various biotechnological applications. For instance, they can be valuable tools in bioremediation projects where preserving existing microbial communities is crucial.

### 3.5. Antioxidant Activity

Microbial BSs/BEs possess a remarkable range of functionalities. Beyond altering surface properties and exhibiting antimicrobial activity, they can also act as antioxidants by blocking oxidative chain reactions [67]. Thus, the DPPH assay was used to investigate the scavenging ability of the gordofactin produced by *G. alkanivorans* strain 1B. The antioxidant activity of the extracted gordofactin (lyophilized sample) was measured using a concentration range from 50 mg/L to 6600 mg/L, and L-ascorbic acid, at a concentration range from 0.3 mg/L to 62.5 mg/L, was used as a standard antioxidant (positive control).

**Figure 5:**
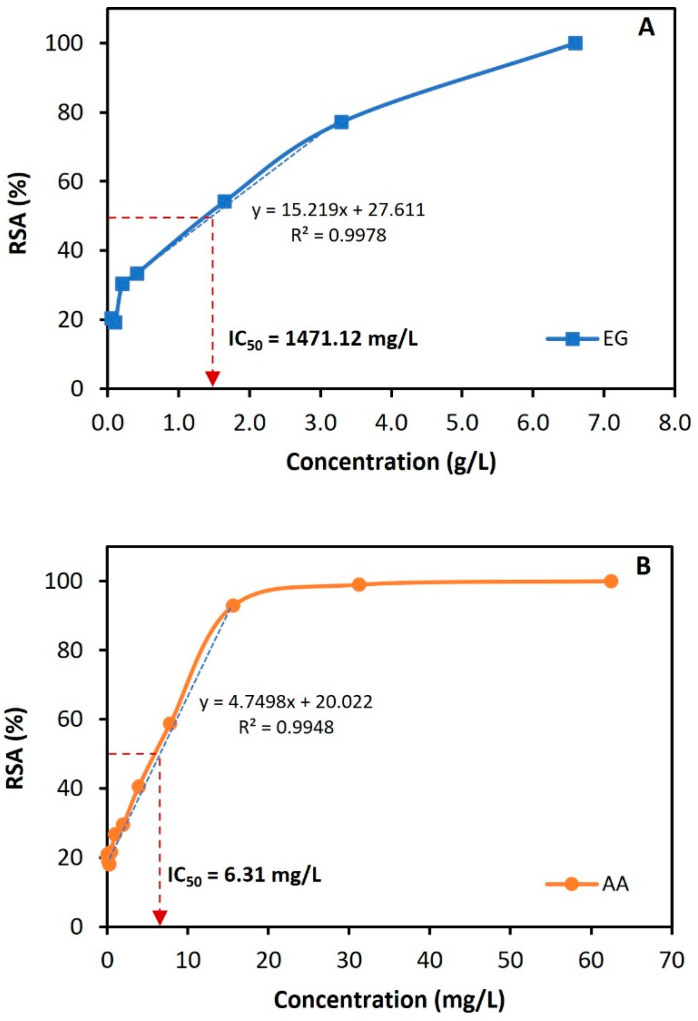
DPPH radical scavenging activity (%) for the extracted gordofactin (**A**), produced by *G. alkanivorans* strain 1B, and the ascorbic acid (**B**), as the positive control. Each data point represents an average of at least 3 replicates (SD ≤ 4.6 ± 0.5%).

Figure 5 presents the plots of the concentration-dependent radical scavenging activity [% RSA = f(concentration)] for gordofactin (Figure 5A) and L-ascorbic acid (Figure 5B), from which the respective IC50 values were estimated. At a concentration of 52 mg/L, the EG only scavenged 20.4% of DPPH radicals, while L-ascorbic acid achieved the same level of scavenging at a much lower concentration (0.49 mg/L). L-ascorbic acid at a concentration of 7.8 mg/L exhibited a percentage of DPPH RSA of 58.8%, and at 62.5 mg/L, 100%, highlighting its high antioxidant activity. The IC_50_ value estimated for L-ascorbic acid was 6.31 ± 0.29 mg/L, which is within the range observed in other studies with this reference antioxidant (0.60–98 mg/L) [68,69,70,71]. The IC_50_ calculated for EG was 1471.12 ± 67.67 mg/L, achieving 100% DPPH radicals scavenging at the concentration of 6600 mg/L (Figure 5A). It is important to point out that the extraction protocol was not optimized to preserve antioxidant activity and mostly considered BS/BE properties. By limiting contact with light or exposure to oxygen, it could be possible to obtain significant improvements. Nonetheless, these results still point to the significant antioxidant capacity of gordofactin. The IC_50_ values quantitatively describe the radical scavenging affinity. The lower the IC_50_ value, the higher the DPPH radical-removing ability of the antioxidant. Indeed, IC_50_ value is widely used in biochemistry to compare the radical scavenging capacities of different antioxidants.

The antioxidant capacity of several lipopeptide, glycolipid, and glycopeptide biosurfactants has been extensively studied. Giri et al. [72] reported maximal DPPH scavenging activities of 69.1% and 73.5% for two lipopeptide biosurfactants isolated from *Bacillus* strains, both at 5 g/L concentrations. In another study, Jemil et al. [73] obtained 80.6% DPPH radical scavenging at a concentration of 1 g/L of lipopeptide biosurfactants from *Bacillus*. For a glycolipid (rhamnolipids) from Pseudomonas aeruginosa, Abdollahi et al. [74] obtained 50% scavenging capacity (IC_50_) of DPPH radical at a concentration of 1980 mg/L (4.15 mM), which is a higher IC_50_ than that observed for EG from *G. alkanivorans* strain 1B (≈1471 mg/L). As for a water-soluble glycopeptide, the IC_50_ of DPPH radical scavenging was found to be 133.5 mg/L [75].

The antioxidant and protective properties of lipopeptide, glycolipid, and glycopeptide biosurfactants against oxidative stress constitute a comprehensive feature that provides them excellent potential as biological alternatives to their chemical counterparts in a wide range of biotechnological and industrial applications. The search for these effective and eco-friendly alternatives has been fueled by the growing awareness of environmental sustainability and potential health risks associated with chemical surfactants, which make these biomolecules particularly attractive for applications in areas such as medicine and cosmetics [76].

## 4. Conclusions

In this study, we successfully characterized, in depth, the physical, biochemical, and functional properties of the lipoglycopeptide gordofactin, a biomolecule with surfactant and emulsifier properties produced by *G. alkanivorans* strain 1B, when cultivated in a continuous bioreactor with fructose as a carbon source. The partially purified gordofactin (EG/LEG sample) effectively reduced surface tension and possessed a critical micelle concentration (CMC) comparable to commercially available surfactants. It displayed remarkable stability and retained emulsifying activity across a broad range of temperatures (30 °C to 80 °C) and pH (pH 3–12). Moreover, a significant tolerance of gordofactin EA to a wide range of NaCl concentrations (1 to 100 g/L) was demonstrated. Although with a great loss of EA in the presence of NaCl concentrations > 2.5%, gordofactin can still tolerate up to 100 g/L NaCl, maintaining about 16% EA for up to 7 days. These promising features emphasize the robustness of gordofactin and make it suitable for high-temperature applications, diverse pH environments, and saline conditions. Furthermore, gordofactin exhibited growth inhibition against both Gram-positive and Gram-negative bacteria, and it demonstrated concentration-dependent free radical scavenging activity (IC_50_ ≈ 1471 mg/L).

These findings highlight the potential of gordofactin as an eco-friendly BS/BE alternative to conventional surfactants/emulsifiers. Indeed, the recent wide use of microbial BSs/BEs in several sectors is escalating market growth. Their surface activity, emulsifying properties, stability, and antimicrobial and antioxidant properties, among other unique characteristics, make them attractive candidates for various applications, including bioremediation, enhanced oil recovery, household detergents, cosmetics and personal care products, the food industry, and pharmaceutical manufacturing. Therefore, delving deeper into these promising surface-active biomolecules, pushing bioprocesses towards novel BSs/BEs, can contribute to the development of a new generation of eco-friendly alternatives for various industrial and biotechnological applications, which may attract new players in the bio-based surfactants/emulsifiers market. In fact, more focus on product differentiation, reducing costs, and supply chain optimization remain crucial for the widespread adoption of microbial BSs/BEs.

## Figures and Tables

**Table 1:** Characterization of gordofactin produced by *G. alkanivorans* strain 1B, in continuous culture, with 20 g/L fructose as the C-source, at sequential stages of its partial purification: cell-free supernatant (crude gordofactin), gordofactin concentrate (from ultrafiltration by 15 kDa/1000 kDa MWCO membranes), and extracted gordofactin, before/after sample lyophilization.

	Cell-Free Supernatant	GC-15	LGC-15	EG	LEG	LGC-1000
Suspended solids (g/L)	8.69 ± 0.50	15.51 ± 0.25		7.36 ± 0.12		
EA (U/mL)	40.0 ± 2.0	144.0 ± 5.0		83.0 ± 5.0		
SEA (U/mg)	4.6	9.3	13.5	11.3	9.5	10.96
CMC (mg/L)			26.7		13.5	29.4

GC-15: gordofactin concentrate from ultrafiltration by the 15 kDa MWCO membrane. LGC-15: lyophilized gordofactin concentrate from ultrafiltration by the 15 kDa MWCO membrane. EG: extracted gordofactin using chloroform–methanol. LEG: lyophilized extracted gordofactin. LGC-1000: lyophilized gordofactin concentrate from ultrafiltration by the 1000 kDa MWCO membrane.

## Data Availability

The authors confirm that the datasets supporting the findings and conclusions of this study are contained within the article.
